# National Survey on Access, Use and Promotion of Rational Use of Medicines: methods

**DOI:** 10.11606/S1518-8787.2017051007027

**Published:** 2017-09-22

**Authors:** Juliana Álvares, Maria Cecilia Goi Porto Alves, Maria Mercedes Loureiro Escuder, Alessandra Maciel Almeida, Jans Bastos Izidoro, Augusto Afonso Guerra, Karen Sarmento Costa, Ediná Alves Costa, Ione Aquemi Guibu, Orlando Mario Soeiro, Silvana Nair Leite, Margô Gomes de Oliveira Karnikowski, Francisco de Assis Acurcio

**Affiliations:** I Departamento de Farmácia Social. Faculdade de Farmácia. Universidade Federal de Minas Gerais. Belo Horizonte, MG, Brasil; II Secretaria Estadual de Saúde de São Paulo. São Paulo, SP, Brasil; III Faculdade de Ciências Médicas de Minas Gerais. Belo Horizonte, MG, Brasil; IV Núcleo de Estudos de Políticas Públicas. Universidade Estadual de Campinas. Campinas, SP, Brasil; V Programa de Pós-Graduação em Saúde Coletiva. Departamento de Saúde Coletiva. Faculdade de Ciências Médicas. Universidade Estadual de Campinas. Campinas, SP, Brasil; VI Programa de Pós-Graduação em Epidemiologia. Faculdade de Medicina. Universidade Federal do Rio Grande do Sul. Porto Alegre, RS, Brasil; VII Instituto de Saúde Coletiva. Universidade Federal da Bahia. Salvador, BA, Brasil; VIII Departamento de Saúde Coletiva. Faculdade de Ciências Médicas. Santa Casa de São Paulo. São Paulo, SP, Brasil; IX Faculdade de Ciências Farmacêuticas. Pontifícia Universidade Católica de Campinas. Campinas, SP, Brasil; X Departamento de Ciências Farmacêuticas. Universidade Federal de Santa Catarina. Florianópolis, SC, Brasil; XI Faculdade de Ceilândia. Universidade de Brasília. Brasília, DF, Brasil

**Keywords:** Pharmaceutical Services, Health Services Research, methods, National Drug Policy, Unified Health System, Assistência Farmacêutica, Pesquisa sobre Serviços de Saúde, métodos, Política Nacional de Medicamentos, Sistema Único de Saúde

## Abstract

The *Pesquisa Nacional sobre Acesso, Utilização e Promoção do Uso Racional de Medicamentos* –*Serviços* (PNAUM – National Survey on Access, Use and Promotion of Rational Use of Medicines – Services) aimed to characterize the organization of pharmaceutical services in the Primary Health Care of the Brazilian Unified Health System (SUS). PNAUM – Services is a cross-sectional and evaluative study, with planned sample of 600 cities, held between 2014 and 2015, composed of a remote phase, with telephone interviews with health managers. Of these 600 cities, 300 were selected for a survey on health services. We selected the 27 capitals, the 0.5% largest cities of each region, and the remaining cities were drawn. The estimate of the representative national sample size considered three levels: cities, medicine dispensing services, and patients. The interviews were carried out with a structured questionnaire specific for: municipal secretaries of health, professionals responsible for pharmaceutical services in the city, professionals responsible for the dispensing of medicines, physicians, and patients. The secondary data were obtained in official databases, in the latest update date. PNAUM – Services was the first nationwide research aimed at the assessment and acquisition of national and regional indicators on access to medicines, as well as use and rational use, from the perspective of various social subjects.

## RESUMO

A Pesquisa Nacional sobre Acesso, Utilização e Promoção do Uso Racional de Medicamentos (PNAUM) – componente Serviços teve por objetivo caracterizar a organização dos serviços de assistência farmacêutica na Atenção Básica do Sistema Único Saúde. A PNAUM – Serviços foi um estudo transversal, avaliativo, com amostra planejada de 600 municípios, realizado entre 2014 e 2015, composto por uma fase remota, com entrevistas telefônicas com gestores. Desses 600 municípios foram selecionados 300 para um inquérito em serviços de saúde. Foram selecionadas as 27 capitais, 0,5% dos maiores municípios de cada região e realizado um sorteio dos demais municípios. O cálculo do tamanho da amostra representativa nacional considerou três níveis: municípios, serviços de dispensação de medicamentos e usuários. As entrevistas foram realizadas com a utilização de um questionário estruturado específico para: secretário municipal de saúde, responsável pela assistência farmacêutica no município, responsável pela entrega de medicamentos, médico e usuário. Os dados secundários foram obtidos em bases oficiais, na data mais recente de atualização. A PNAUM – Serviços foi a primeira pesquisa de âmbito nacional visando a avaliação e obtenção de indicadores nacionais e regionais acerca de acesso a medicamentos, bem como utilização e uso racional, sob a ótica de variados atores sociais.

## INTRODUCTION

Pharmaceutical Services (PS) are one of the components of the Brazilian Unified Health System (SUS), fundamental to its principle of care integrality. Even before they were established as a public policy^3^, they implemented the access and the rational use of medicines in their conception.

In the search for strengthening and consolidation of PS in Brazil, the *Política Nacional de Medicamentos* (PNM – National Drug Policy)^4^ was approved in 1998 and, later, the *Política Nacional de Assistência Farmacêutica* (PNAF – National Policy of Pharmaceutical Services), in 2004.

Studies indicate the existence of a big gap between the legally established PS and the real primary PS of Brazilian cities. The problems range from the lack of essential medicines and bad conservation in the storage process to the total absence of guidance of proper use to the patient^6^.

Studies on the use, access, and rational use of medicines in national scale provide subsidies to governmental authorities to assess their policies of PS, investments in the selection and acquisition of medicines, as well as control of expenses.

Furthermore, it is essential that evaluative researches in the area of pharmaceutical services address the many issues related to the system as a whole, to the adequacy of services to the real needs of the patients, and that these people, in turn, be perceived as central subjects of the process.

In this perspective, the *Pesquisa Nacional sobre Acesso, Utilização e Promoção do Uso Racional de Medicamentos* (PNAUM – National Survey on Access, Use and Promotion of Rational Use of Medicines) was developed, established by Ordinance of the Brazilian Ministry of Health no. 2,077, September 17, 2012^5^. PNAUM has been performed by two strategies, both with national scope: a household survey and a component of services. PNAUM – Services aimed to characterize the organization of PS in the Primary Health Care of SUS, focusing on the access and promotion of the rational use of medicines, as well as to identify and discuss factors that interfere in the consolidation of PS in the Primary Health Care of the cities.

This article presents the methodology of PNAUM – Services, with details of the route and of the methodological procedures adopted in the planning and execution of the field research.

## METHODS

PNAUM – Services is a cross-sectional and exploratory study of evaluative nature. To respond to its goals, three thematic axes were conceived:

- Municipal management of PS, covering topics such as formal structure and organization of municipal pharmaceutical services, financing and investment in pharmaceutical services, acquisition of medicines, health care waste management, mechanisms for popular participation and receiving of criticism and suggestions on management, information on the workforce that operates in pharmaceutical services, medicine selection and standardization, organization of places of storage and dispensing of medicines, advertising regulations and entry of representatives of pharmaceutical industries in primary health care services;
- Operationalization of PS, including topics such as knowledge of the organization of PS in the city (focusing on the list of medicines and protocols, as well as prescribing habits, including non-standard medicines), professional/patient relation, notification of technical complaints and adverse events, evaluation of the organization of PS, political interference in PS and/or in the dispensing of medicines;
- Consumption, with themes such as the most used medicines by patients and reasons of use, self-medication, offer of free samples, perception about prescription tracking and non-medicated guidelines, profile of the patients of the services, users’ perception about the existence of predefined tracer diseases, use and characteristics of care in the health system, medicine acquisition process, aspects related to the use and rational use of medicines, as well as to the quality and lifestyle.

Based on the main themes, the sampling plan was delineated (delimited to the study populations), and the processes of data collection were set.

### Sampling plan

In the sampling plan, the following study populations were considered: municipal secretaries of health, professionals responsible for pharmaceutical services in the cities, medicine dispensers, physicians, and patients of health services.

We opted for the use of sampling in various stages of selection. In each stage, one or two of these populations were sampled and the estimates relating to them were made separately^2^. Thus, in the drawn cities, secretaries of health and those responsible for pharmaceutical services were accessed (two people from each cities, except when there was accumulation of both functions) and in health services, those responsible for the dispensing (one person per service). In these services, patients and physicians were also sampled (Table 1).

**Table 1:** Axes of analysis, study populations, drawing unit, and elements of the sample. National Survey on Access, Use and Promotion of Rational Use of Medicines – Services, 2015.

Axis of analysis	Study population	Drawing unit	Elements included in the sample
Management	Secretaries of Health	City	One Secretary in each city
	Professionals responsible for pharmaceutical services	City	One professional in each city
Operationalization and consumption	Professionals responsible for dispensing of medicines	City and Health Service	One professional in each health service
	Physicians	City and Health Service	Physicians present in the days of research
Consumption	Patients	City, Health Service, and Medical appointment	Patients in appointment drawn in the days of research

Source: PNAUM – Services, 2015.

Therefore, three samples were drawn: of cities, services, and patients. In the first, the cities were the single drawing units, since there is only one secretary of health and one person responsible for PS in each city, in a procedure equivalent to the sampling of elements. In the second, these cities became conglomerates (primary sampling units), in which the services that composed the sample were drawn. And in the third, the services became secondary sampling units, in which the patients were drawn.

The study populations were stratified per region: North, Northeast, South, Southeast, and Midwest, and these strata constituted the study domains.

Considering the goal of estimating various proportions, the sample size was calculated by using the algebraic expression: $n_{0}=\frac{P\left(1-P\right)}{{\left(d/z\right)}^{2}}$, where P = 0.50 is the ratio of elements (secretaries of health, professionals responsible for pharmaceutical services, professionals responsible for the dispensing of medicines, and patients) to be estimated as it is the one that leads to greater sample size; z = 1.96 is the normal curve value reduced to the level of confidence of 95% of the confidence intervals; *deff* is the design effect, and *d* is the sampling error in percentage points^2^. The sample sizes adopted in each region were: 112 secretaries of health/professionals responsible for pharmaceutical services in the cities (*deff* = 1.2 and *d* = 0.10); 300 professionals responsible for the dispensing of medicines in health services (*deff* = 2 and *d* = 0.08), and 534 patients (*deff* = 2 and *d* = 0.06), values rounded to 120, 300, and 540. Compared with the sample of patients, the need to get estimates for smaller subgroups was considered and thus the sample of 1,800 patients (n = 540/0.30) was planned. We considered, therefore, groups that included at least 30% of the entire population as the object of analysis, maintaining the desired precision.

Regarding physicians, we planned to include in the sample the ones present in the days of data collection of patients (estimated based on the size of the patient sample), for believing that the number of physicians present in the services is proportional to the number of patients. The number of physicians in the services should be noted, if necessary the introduction of weights.

To compose the sample of cities, 120 cities were drawn in each region, in which should be interviewed the secretaries of health and the professionals responsible for pharmaceutical services The list used to draw the cities was extracted from the *Departamento de Informática do SUS* (Datasus – Department of Informatics of SUS), with the resident population estimated for 2012 by the Brazilian Institute of Geography and Statistics (IBGE).

To ensure the inclusion in the sample of all the capitals and cities considered large in each region, the following strata were constituted: capitals; larger cities (the 0.5% of the largest cities in the region), and smaller cities. The first two were considered certain strata, in which there was no drawing of units. In the stratum of smaller cities, the selection was carried out by systematic draw, with the cities ordered by population size.

The sampling fraction^1^ corresponding to the draw of cities, in each stratum, was: $f=\frac{a}{A}$, where *a* is the number of cities in the sample and *A*, the number of existing cities. In the first two strata (capital and larger cities) the sampling fraction corresponded to the unit.

The sample of health services, in which medicine dispensers should be interviewed, was drawn in two stages^1^: cities and services. In the first stage, 60 cities were drawn from the 120 cities selected previously. The total of the capitals and larger cities and of other cities was taken; the remaining number to complete 60 was drawn among the smaller cities.

In the second stage, in each city, health services relating to primary health care facilities were drawn, registered in the *Cadastro Nacional de Estabelecimentos de Saúde* (CNES – National Registration of Health Establishments) as being of the following types: health clinic, health center or basic health unit, and mixed unit. River and land mobile units were excluded.

For the draw, the small cities were divided into two groups: with one or two health services and with three or more health services. In the first group, with one or two services, there was no draw. The services were distributed in the strata settling 100 services in stratum 1 (capitals), to obtain separate estimates for the capitals.

The sampling fraction used was: f=aA·bB , where *a* and *A* are, respectively, the numbers of cities in the sample and in the population; b̄ and B̄ are the averages per city of services in the sample and in the population, estimated by b and B , being *B* the number of services in the cities of the sample.

For the draw of the sample of physicians and patients, sampling was used in three stages: city, health service, and physician or patients.

We established that 1,800 patients would be interviewed per region of the country. Considering the occurrence of a non-response percentage of 15% (refusal, impossibility of carrying out the full interview etc.), 2,100 patients were drawn. In each region, this number of patients was proportionally distributed by the strata, according to the frequency of services sampled in each of them.

The sampling fraction used to draw patients in services was: f3=cC where c̄ is the average of patients drawn by service, given by the ratio between the total number of patients and the number of sample services, and C̄ is the daily average of patients seen in medical appointment in the services, estimated by:C=Csns , where C_*s*_ is the total of monthly appointments in the health services of the sample, and n_*s*_ is the number of services.

For this estimation, we used data regarding the medical appointments made per service from July to December 2013 and in May 2014 in people over the age of 17 years, according to list provided by the Secretariat of Primary Health Care of the Brazilian Ministry of Health.

The denominators of the sampling fractions in the services 1c/C for each stratum and region corresponded to the drawing interval in the services. Thus, by applying these fractions to the number of patients in each service, the numbers of patients to be interviewed in these services were determined.

For patients, the global sampling fraction was: f=aA·bB·cC=nN

The drawn of patients in each service cannot be performed from lists of patients, as it would be expected in probability sampling. We opted to establish criteria for the selection of patients that would not allow interviewers to choose patients to compose the sample, approximating the selection to a random drawing.

Interviewers were instructed to verify the number of interviews to be conducted in each service and estimate the number of days required to complete these interviews. In the health service, after obtaining the consent of the coordinator of the service for the collection of data required for research, the names of all physicians should be surveyed in each day that the interviewer remained in the service. The names would be listed alphabetically in the planning worksheet of the draw and the interviews would be distributed by the physicians of the worksheet according to the alphabetical order. After this stage, the first patient should be identified for interview on the agenda of any physician of the sample: it would be the last patient to be seen among those who were already present in the service.

Regarding physicians, those who were in the service in the days of interviews should be included in the sample.

As a result of the use of different probabilities of selection in the draw of the sample units, the estimates obtained in this study were weighed and the weight of each research unit corresponded to the inverse of its sampling fraction.

Table 2 shows the sampling plan according to strata.

**Table 2:** Sample planned according to strata. National Survey on Access, Use and Promotion of Rational Use of Medicines – Services, 2015.

Sample	Unit	Region	Capitals	Larger cities	Cities		Total
Cities	Cities	North	7	2	111		120
		Northeast	9	9	102		120
		Southeast	4	8	108		120
		South	3	6	111		120
		Midwest	4	2	114		120
					1e2ss^a^	3e+ss^b^	
Services	Cities	North	7	2	20	31	60
		Northeast	9	9	8	34	60
		Southeast	4	8	17	31	60
		South	3	6	28	23	60
		Midwest	4	2	23	31	60
	Services	North	100	25	27	142	295
		Northeast	100	29	13	172	315
		Southeast	100	27	24	161	312
		South	100	27	34	152	313
		Midwest	100	22	32	152	306
Patients	Patients	North	712	181	192	1,013	2,100
		Northeast	667	195	87	1,150	2,100
		Southeast	673	185	162	1,083	2,100
		South	671	180	228	1,018	2,100
		Midwest	686	150	220	1,044	2,100

^a^ Cities with 1 or 2 health services

^b^ Cities with 3 or more health services

Source: PNAUM – Services, 2015.

### Processes for Data Collection

We adopted, for data collection, surveys *in loco* in the primary health care services selected randomly, by applying direct observation guidelines and interviews (patients, professionals responsible for the dispensing of medicines, and prescribers), as well as telephone interviews with municipal health managers using semi-structured questionnaires. Also, we performed an investigation of data from secondary sources, to characterize the cities.

The interviews were carried out using a structured questionnaire for each study population: municipal secretary of health, professional responsible for PS in the city, professional responsible for the dispensing of medicines, physician, and patient. The interviews lasted 30 minutes on average. However, for patients, this time varied according to the number of comorbidities presented and medicines used. Personal information were collected from all respondents, in addition to the specific questions of the study.

The questionnaires for the municipal secretaries of health and for the professionals responsible for pharmaceutical services were applied by telephone interview. Questionnaires for physicians were self-applicable and questionnaires for patients and dispensers of medicines were applied in person in the primary health care services.

The interview with the professional responsible for dispensing medicines was held at the health unit, pharmacy, or dispensary. And, finally, the interviews with patients were carried out in the primary health care services, most of the time before the medical appointment. All questionnaires presented open-ended questions to evaluate the performance and factors related to the functioning of the health services.

### Observation and Photographic Record of the Services of Dispensing of Medicines

The observation guideline with photographic record was used to evaluate pharmacies/public dispensing units of medicines for the primary health care, being them either inside or outside the health services. The places of storage and dispensing were observed, to investigate the conditions under which the dispensing or delivery of medicines is performed, in addition to the fractionation and storage conditions, the record of the activities, the physical availability of the medicines selected, the existence of expired medicines, and the management of medicines unsuitable for use.

After collecting information about the operation, registration, and infrastructure of the unit, the observer photographed different areas: counter/balcony/window of dispensing of medicines, internal area of the pharmacy, pharmacy inventory area (if there was any), medicines’ shelves (panoramic and with proximity to identification of products, from the place of storage of controlled medicines). The observer also obtained copies, when available, of the standard operating procedures used in the dispensing sector.

After the development of the research instruments, we defined the flowchart of the field research, to guide and standardize the conduct of the interviewers. The main stages of the study are presented in the Figure.

**Figure:**
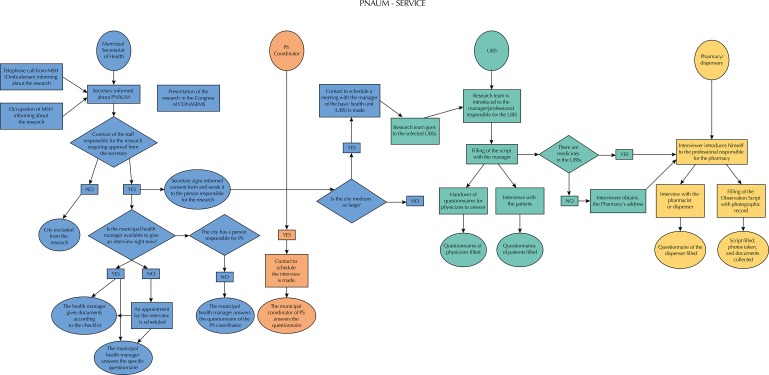
Flowchart of the field research of the National Survey on Access, Use and Promotion of Rational Use of Medicines – Services, 2015.

PS: pharmaceutical services

Source: PNAUM – Services, 2015.

A manual for each instrument of research and a glossary of technical terms were developed. After the stages of elaboration and alignment, a pretest was carried out, involving the cities of different sizes of populations: a capital of a federal unit of the country, a large city, and a small one. The objective, during the *in loco* application, was to discover potential problems and to identify opportunities to improve and adjust the instruments.

Interviewers were trained and informed about the fundamental aspects of the research and standardization of data collection procedures. The whole interview was preceded by information to the person interviewed about the objectives of the research and by the signature of the informed consent form. Research results will be published in aggregate form, ensuring the anonymity of participants.

PNAUM was approved by the National Research Ethics Committee of the National Health Council, under Opinion no. 398,131/2013. All participants signed the informed consent form.

### Secondary Data

The secondary data were obtained on official databases, according to their availability and considering the latest update date. We consulted IBGE, DATASUS [*Sistema de Informação sobre Mortalidade* (SIM – Mortality Information System); *Sistema de Informação sobre Nascidos Vivos* (SINASC – Live Birth Information System); *Sistema de Informações Hospitalares do SUS* (SIH/SUS – Hospital Information System of SUS); *Sistema de Informação de Agravos de Notificação* (SINAN – Notifiable Diseases Information System); *Sistema de Informação da Atenção Básica* (SIAB – Primary Health Care Information System)], *Sistema de Informações sobre Orçamentos Públicos em Saúde* (SIOPS – Information System on Public Health Budgets), and CNES. Also, we evaluated, when available, the municipal health plan and the agreements of the intermanagement committees (bi- and tripartite).

Secondary data included indicators of demographic profile of each city and state; socioeconomic profile; mortality indicators; morbidity indicators; vaccination coverage; prenatal coverage; quantity of professionals and services in the cities; characterization of the municipal health system (information focused on sanitary surveillance, coverage and legal questions of management, such as reports and contracts). They also included other secondary information related to PS, such as the percentage of *Núcleos de Apoio à Saúde da Família* (NASF – Family Health Support Centers) with a pharmacist in the city; total number of public establishments with pharmacy or medicine dispensing units in the city; medicines and standardized protocols; agreement, existence, and normative aspects about the Pharmacy and Therapeutics Committee and municipal health plan; funds received and invested in pharmaceutical services in different components and in the popular pharmacy program; accountability; folders, newsletters, and campaigns; laws, ordinances, or resolutions concerning the area.

## CONCLUSIONS

This is the first nationwide research focused on access and use of medicines in Brazil. With a well designed sampling plan, our intention is to enable analyses related to the various dimensions of access and the study of indicators of rational use and degree of following of prescriptions in relation to adherence to treatment and care with high-prevalence diseases, such as hypertension and diabetes.

Thus, we expect that the results of PNAUM can contribute to the improvement of health policies and to the consolidation of SUS, aiming to improve health conditions and, consequently, the quality of life of the Brazilian population.
